# Impact of disrupted cyclic stretch in intracranial aneurysms: Insights from endothelial cell transcriptomic dataset

**DOI:** 10.1016/j.dib.2023.110014

**Published:** 2023-12-28

**Authors:** Mannekomba R. Diagbouga, Sylvain Lemeille, Sandrine Morel, Brenda R. Kwak

**Affiliations:** a Department of Pathology and Immunology, Faculty of Medicine, University of Geneva. Rue Michel-Servet 1, 1211 Geneva, Switzerland; b Geneva Center for Inflammation Research, Faculty of Medicine, University of Geneva. Rue Michel-Servet 1, 1211 Geneva, Switzerland; c Division of Neurosurgery, Department of Clinical Neurosciences, Faculty of Medicine, Geneva University Hospitals and University of Geneva. Rue Gabrielle-Perret-Gentil 4, 1211 Geneva, Switzerland

**Keywords:** Cyclic mechanical strain, Endothelium, RNA-seq, Cell junctions

## Abstract

Intracranial aneurysm (IA) rupture is a common cause of hemorrhagic stroke. The treatment of unruptured IAs is a challenging decision that requires delicate risk stratification. The rate of poor clinical outcomes after surgical intervention (aneurysm clipping) or endovascular coiling remains elevated (6.7% and 4.8%, respectively), and they do not provide an absolute guarantee to prevent IA growth and rupture. Currently, there is no pharmaceutical treatment to cure or stabilize IAs. Improving the current or developing new treatments for IA disease would require a better understanding of the cellular and molecular mechanisms occurring in the different stages of the disease.

Hemodynamic forces play a critical role in IA disease. While the role of wall shear stress in IAs is well-established, the influence of cyclic circumferential stretch (CCS) still needs clarification. IAs are generally characterized by a lack of CCS. In this investigation, we sought to understand the effect of aneurysmal CCS on endothelial cell (EC) function and its potential significance in IA disease, hypothesizing that CCS can influence IA wall remodelling. RNA-seq data were generated from human umbilical vein ECs (HUVECs) exposed to physiological (6%) or aneurysmal CCS (static). We performed differential gene expression and pathway enrichment analysis. Additionally, we highlighted cell junction gene expression between static and 6% CCS to contribute to the debate about how cell junctions affect endothelium stability and integrity. Researchers in the vascular biology field may benefit from this transcriptomic profile to understand the effect of mechanical stretch on EC biology and its potential significance in vascular disease development.

Specifications Table

**Table:** 

Subject	Biological Sciences/ Cell and Molecular biology
Specific subject area	Endothelial cell gene expression in response to physiological and aneurysmal cyclic stretch.
Data format	Raw (Fastq), Analyzed (Raw counts), Filtered (Differential Gene Expression)
Type of data	Table, Graph, Figure
Data collection	Human umbilical vein endothelial cells were subjected to 48 h of physiological or aneurysmal cyclic stretch using the Flexcell strain unit FX-5000T. The success of the stretch was assessed by checking eNOS phosphorylation on serine 1177 through Western Blot analysis. RNA was isolated, and sequencing was performed using Illumina HiSeq 4000. Validation experiments were conducted using Western Blot and quantitative RT-PCR analysis. Statistical analyses were performed using GraphPad Prism and R software.
Data source location	• **Institution**: Department of Pathology and Immunology, Faculty of Medicine, University of Geneva • **City**: Geneva • **Country**: Switzerland • **Geographical coordinates**: 46.1935° N, 6.1512° E
Data accessibility	Repository name: GEO database, Mendeley Data identification number: GSE222834, doi:10.17632/wkxnrfbzzc.2 Direct URL to data: https://www.ncbi.nlm.nih.gov/geo/query/acc.cgi?acc=GSE222834 https://data.mendeley.com/datasets/wkxnrfbzzc/1 For peer review only Token for accessing the data: 1. The following secure token has been created to allow review of record GSE222834 while it remains in private status: odmbkiwqrdqntkl. 2. https://data.mendeley.com/datasets/wkxnrfbzzc/1

## 1 Value of the Data

• Our data provides insights into the potential mechanisms involved in the pathophysiology of intracranial aneurysms. Understanding the genetic and pathway alterations in response to aneurysmal cyclic circumferential stretch contributes to the knowledge base of intracranial aneurysm disease.
• We provide an initial framework for delving into specific genes or molecular pathways associated with intracranial aneurysm disease. Other researchers can build up on this work for further research and experimentation.
• Endothelial cells play a crucial role in vascular health, and understanding how mechanical stretch influences their behaviour can have broader implications for vascular biology and disease.
• The focus on cell junction gene expression in the context of physiological and aneurysmal cyclic stretch contributes to ongoing debates about how hemodynamic forces impact cell junctions and influence endothelium stability and integrity.

## 2 Background

The pathogenesis of intracranial aneurysm (IA) is highly complex [1]. Several studies emphasized the role of wall shear stress (WSS) in endothelial cell (EC) dysfunction and IA initiation and growth [2], [3], [4], [5], [6]. Recently, we showed that disturbed endothelial junction organization and subsequent increased permeability in primary cilia-deficient cells may explain the severity of IA disease observed in polycystic kidney disease patients [7]. This work highlighted the importance of endothelium integrity in IA disease. Besides WSS exposure, ECs are also influenced by cyclic circumferential stretch (CCS), the level of which varies along the vascular tree. The elastic human aorta diameter undergoes about 10% increase under physiological conditions, whereas muscular peripheral arteries undergo only approximatively 5% cyclic stretch [8]. Physiological CCS participates in vascular maintenance by regulating processes like cell proliferation, morphology [9], [10], [11] and extracellular matrix (ECM) formation and orientation [12,13]. The levels of CCS in saccular IAs are unknown but generally considered low or even absent. Earlier *in vitro* studies reported that in comparison to no-stretch control condition, the combination of CCS and oscillatory low WSS markedly increased the expression of the gap junction connexin43 in bEnd.3 cells [14] and HUVECs [15], suggesting a relationship between CCS and junction genes. Unravelling the role of cyclic stretch in IA disease requires further research.

## 3 Data Description

### 3.1 RNA sequencing, data quality assessment and differential gene expression

**Fig. 1:**
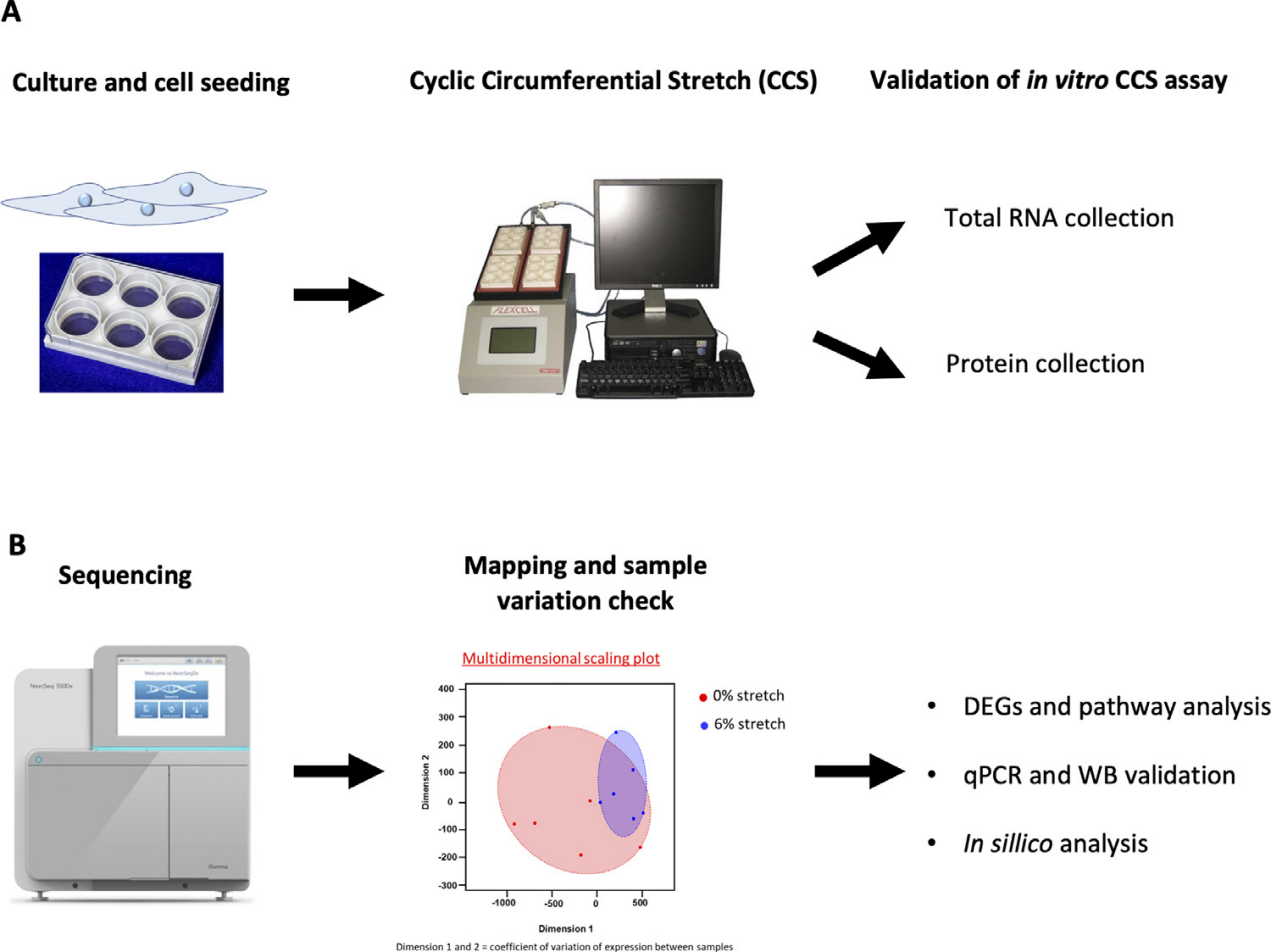
Experimental design and workflow. (A) Human umbilical vein endothelial cells (HUVECs) from 6 different donors were used. Cells were seeded on BioFlex culture plates (left) and submitted to cyclic stretch of 6% or not (0%) for 48 h using the Flexcell strain unit FX-5000T (middle). Protein lysates were collected for Western blot validation experiments. Total RNA was collected for sequencing and qPCR validation. (B) RNA, libraries and sequencing reads quality control have been performed prior to differential expression of genes (DEGs) and pathway analyses.

**Fig. 2:**
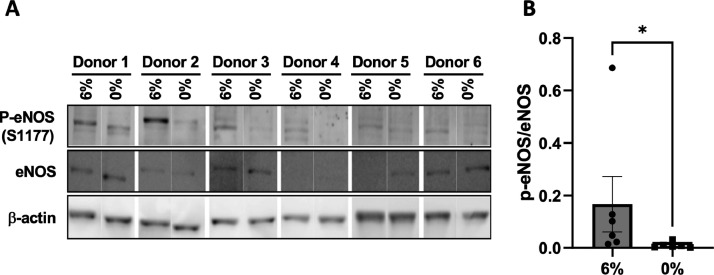
Total and phosphorylated eNOS (Serine 1177) expression. (A) Phosphorylated eNOS (Serine 1177) and total eNOS expression in HUVECs exposed to physiological (6%) or aneurysmal (0%) CCS was assessed by Western blotting. Beta-actin was used as a loading control. (B) Graph shows the levels of phosphorylated eNOS normalized to total eNOS. Differences between groups were analysed using a ratio paired *t*-test. *p < 0.05.

**Fig. 3:**
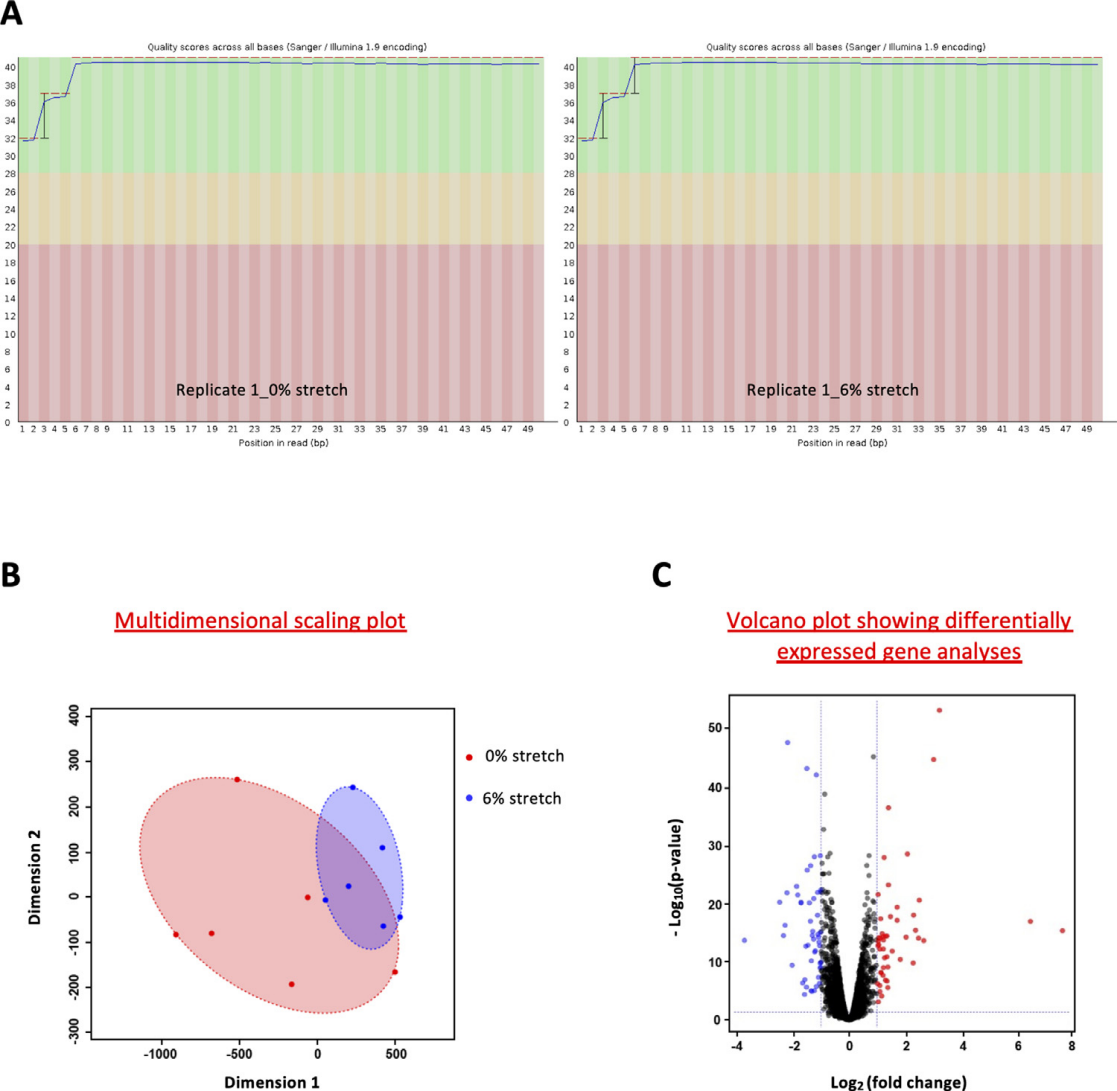
Quality assessment and differential expression analysis. (A) Base quality along the length of the reads in replicate 1 submitted to 0% stretch (left) and 6% stretch (right) as a representative sample. (B) Multidimensional scaling plot representing similarities of gene expression between HUVECs exposed to aneurysmal (0%, red dots) or physiological CCS (6%, blue dots). *N* = 6 for each condition. (C) Volcano plot displaying differential expressed genes between HUVECs exposed to aneurysmal or physiological CCS. The y-axis corresponds to the mean expression value of -log_10_ (*p*-value), and the *x*-axis displays the log_2_ (fold change) value. The red and blue dots represent respectively the up- and down-regulated genes (fold change≥2;*p* < 0.05). (For interpretation of the references to color in this figure legend, the reader is referred to the web version of this article.)

In the present study we compared by next generation RNA-sequencing the transcriptome profile of human umbilical vein ECs (HUVECs) exposed to 48 h of physiological (6%) or aneurysmal (0%) CCS (Fig. 1) using a FlexCell device. Six biological replicates were used to produce high confidence data. As a quality check of stretch assay, we tested whether we could observe in our experiments a well-known response of ECs to CSS, *i.e.* phosphorylation of endothelial nitric oxide synthase (eNOS) on Serine 1177 (Ser1177) [16]. By Western blot, we confirmed a higher level of eNOS phosphorylation on Serine 1177 in HUVECs exposed to physiological CCS (6%) in comparison to cells exposed to aneurysmal CCS (0%), thus validating our methodology (Fig. 2A-2B). Next, we performed RNA sequencing and Fastq, and Raw count data have been submitted to GEO database under accession number GSE222834. The sequencing quality of all samples was assessed with FastQC (Fig. 3A). Table 1 summarizes the number of reads mapping and assigned status. All samples were of high quality. Variation between samples and experimental groups were measured using a multi-dimensional scaling plot (Fig. 3B). We observed some variation between samples under aneurysmal (0%) CCS that lessens considerably when exposed to physiological (6%) CCS.

**Table 1:** RNA-seq raw data and mapping metrics. The reads that are mapped only once to the genome were considered for the read allocation to genomic features. This step was done using featureCounts which counts spliced reads only once and removes reads overlapping two exons from different genes (ambiguous reads). Reads without overlap with Unique Gene Model items are not counted (noFeatures reads).

	Counts	Mapping status			Allocation status		
Sample	Initial	Uniquely mapped	Multiple mapped	Unmapped	Unassigned ambiguity	Unassigned noFeatures	Assigned
static_rep1	33,272,929	28,956,808	4,239,274	76,847	560,227	2,448,607	25,947,974
static_rep2	33,483,044	29,210,081	4,182,496	90,467	581,437	2,482,428	26,146,216
static_rep3	32,238,376	28,151,741	4,014,256	72,379	563,703	2,287,851	25,300,187
static_rep4	30,484,985	26,633,555	3,760,661	90,769	549,249	2,215,912	23,868,394
static_rep5	29,817,619	26,217,189	3,534,906	65,524	515,658	2,339,483	23,362,048
static_rep6	31,878,600	27,815,267	3,992,320	71,013	563,632	2,311,158	24,940,477
stretch_rep1	33,093,926	29,010,825	4,001,600	81,501	556,157	3,091,584	25,363,084
stretch_rep2	31,449,360	27,633,367	3,732,984	83,009	534,295	3,130,656	23,968,416
stretch_rep3	29,973,030	26,282,924	3,614,268	75,838	506,902	2,737,490	23,038,532
stretch_rep4	34,062,413	29,810,060	4,158,369	93,984	571,194	3,041,291	26,197,575
stretch_rep5	33,911,102	30,109,065	3,704,124	97,913	566,816	3,383,125	26,159,124
stretch_rep6	35,466,289	30,946,714	4,435,088	84,487	599,260	3,244,349	27,103,105

The differential gene expression of ECs exposed to physiological or aneurysmal CCS revealed 49 up-regulated genes and 51 down-regulated genes in absence of CCS (Fig. 3C, Supplementary Table 1 (https://data.mendeley.com/datasets/wkxnrfbzzc/1)). Gene set enrichment analysis (GSEA) identified 69 up-regulated and 37 down-regulated pathways in HUVECs exposed to aneurysmal CCS (Supplementary Table 2). Up-regulated pathways were involved in oxidative stress, angiogenesis and inflammation, and the down-regulated pathways in proliferation and ECM-receptor interactions (Supplementary Table 2 (https://data.mendeley.com/datasets/wkxnrfbzzc/1)).

### 3.2 Junction genes expression

**Fig. 4:**
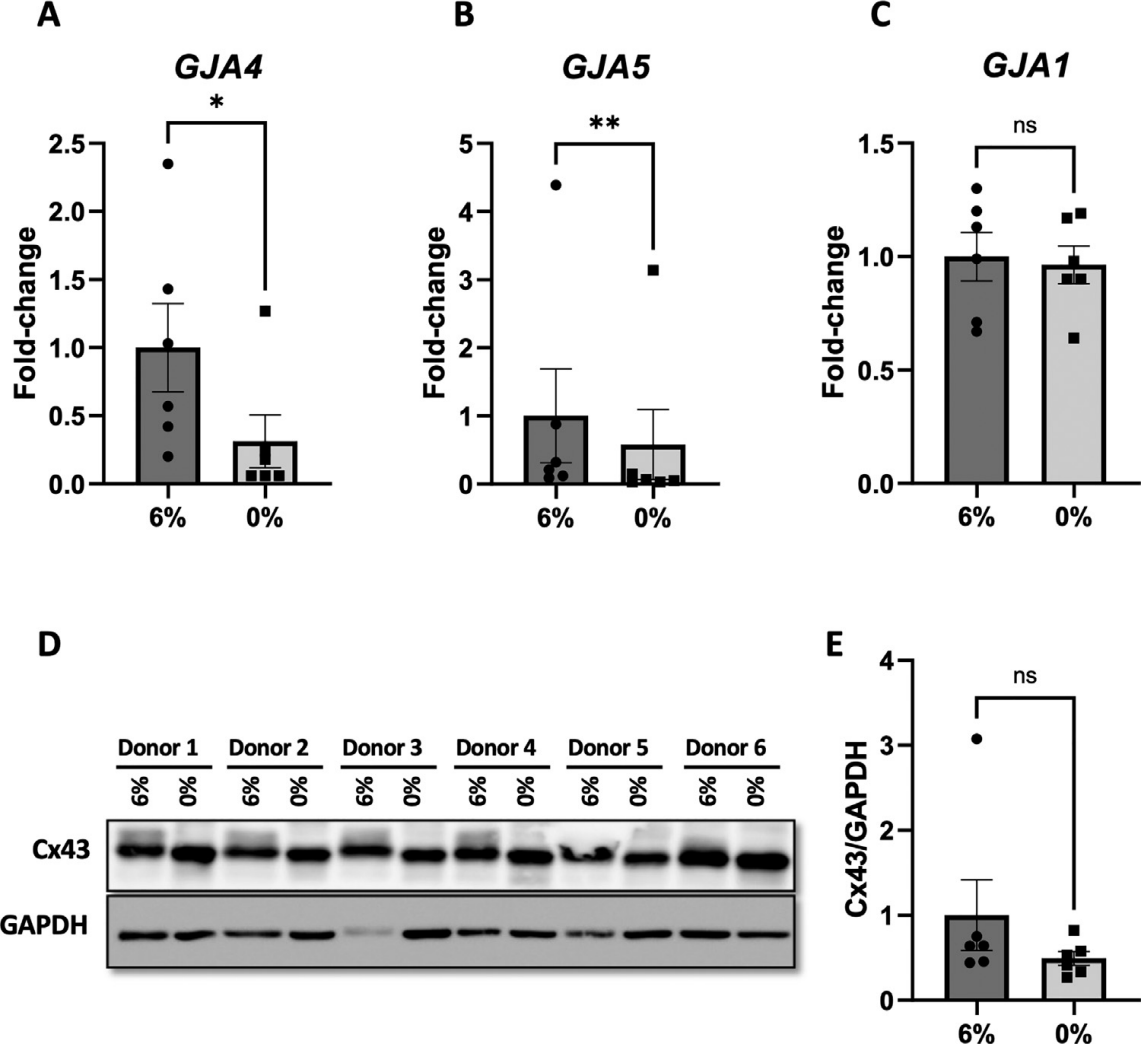
qPCR and Western blot validation. mRNA expression level of (A) *GJA4* (Cx37), (B) *GJA5* (Cx40) and (C) *GJA1* (Cx43) in HUVECs exposed to physiological (6%) or aneurysmal (0%) CCS. (D) Cx43 expression in HUVECs exposed to physiological (6%) or aneurysmal (0%) CCS was assessed by Western blot. (E) Graph shows the levels of Cx43 normalized to GAPDH. Differences between groups were analysed using a ratio paired *t*-test. *p < 0.05, **p < 0.01, ns=non-significant.

**Fig. 5:**
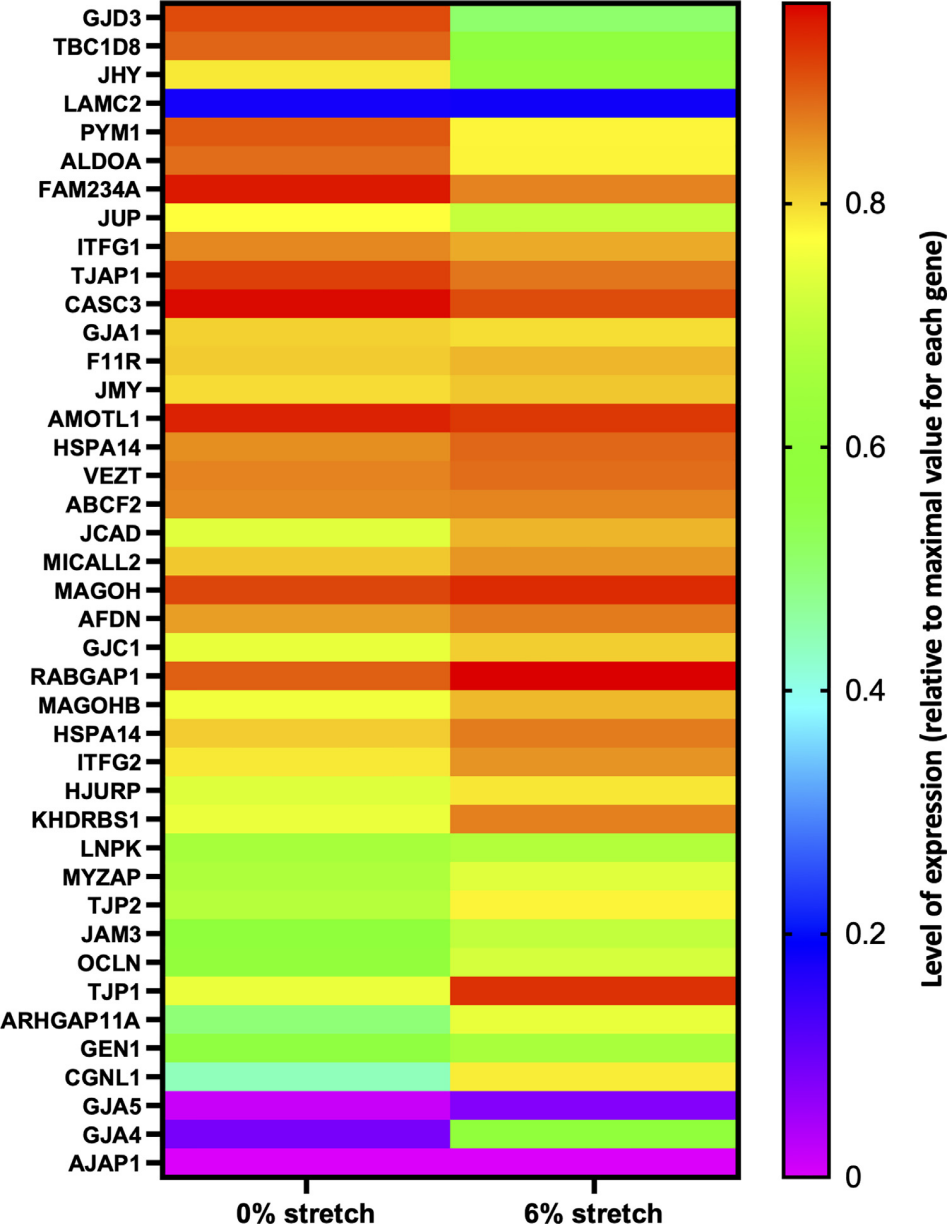
Heatmap showing the expression of Tight, Adherens and Gap Junction genes. The list of junction genes has been retrieved from the HUGO Gene Nomenclature Committee resource. Shown are genes with more than 1 Read per kilobase of exon per million reads mapped. Expression levels are represented as the ratio to the maximal value for each gene.

The RNA-seq analysis revealed that the expression of *GJA4* and *GJA5* genes coding for the gap junction proteins connexin37 (Cx37) and Cx40, respectively, was down-regulated by aneurysmal CCS (LogFC = −2.477 and −2.032, Supplementary Table 1 (https://data.mendeley.com/datasets/wkxnrfbzzc/1)). This down-regulation was confirmed by quantitative PCR (qPCR) in independent experiments (Fig. 4A-3B). Cx43 mRNA expression levels (*GJA1*, Fig. 4C) as well as Cx43 protein expression (Fig. 4D-4E) were similar under physiological and aneurysmal CCS. However, Western blot analysis showed that the electrophoretic mobility of Cx43 was consequently different between 6% and 0% CSS. Indeed, while multiple bands were observed between 41 kDa and 45 kDa under physiological CCS (6%), the signal coalesced into 1 band at 41 kDa under aneurysmal CCS (0%) (Fig. 4D). It is thus likely that physiological phosphorylation of Cx43 is disrupted by absence of CSS. Connexins are crucial proteins for endothelial homeostasis, vascular function, EC cycle regulation and inflammation. Further investigations may help to better define the role of connexins and other junction proteins in IA disease, and to understand the significance of absence of CCS for IA disease progression. Along these lines, and based on the list of junction genes from the HUGO Gene Nomenclature Committee (HGNC), we generated a heatmap highlighting junction gene expression in presence or not of cyclic stretch (Fig. 5).

## 4 Experimental Design, Materials and Methods

### 4.1 Cell culture

Single donor batches of Human Umbilical Vein Endothelial cells (HUVECs) were purchased at Lonza (C2517A). HUVECs from six different donors were grown in dishes coated with 1.5% gelatin (Sigma-Aldrich) at 37°C in a humidified atmosphere containing 5% CO_2_. Cells were cultured in EC Growth Medium (EGM)−2 (C-22,111, PromoCell), consisting of basal medium and supplement pack. The supplement composition and final concentrations after addition to the medium are: fetal calf serum 0.02 ml/ml, epidermal growth factor (recombinant human) 5 ng/ml; basic fibroblast growth factor (recombinant human) 10 ng/ml; insulin-like growth factor (long r3 igf, recombinant human) 20 ng/ml; vascular endothelial growth factor 165 (recombinant human) 0.5 ng/ml; ascorbic acid 1 µg/ml; heparin 22.5 µg/ml; hydrocortisone 0.2 µg/ml. 10,000 U/mL penicillin-streptomycin (Gibco 15,140–122) was further added to the supplemented media. Cells were used at passages 5–6.

### 4.2 Cyclic stretch exposure

HUVECs were seeded at 300′000 cells/well on pre-coated Collagen I BioFlex culture plates® (Flexcell International) and grown until confluence. Then, the cells were submitted to cyclic stretch of 6% or not (0%) for 48 h using the Flexcell strain unit FX-5000T (Flexcell International) according to the manufacturers’ instructions (Fig. 1A). Thus, the cells were stretched over a 25 mm loading station or maintained under static conditions (0%) by placing a Flexstop beneath the well of the BioFlex culture plate. The mechanical stretch was applied following arterial pulse waveform with a frequency of 1 Hz. After 48 h, we collected the cells that received a uniform strain, *i.e.* those in the middle of the well. The area of uniform strain was determined using the following formula: Diameter = (Diameter of loading station)/(1 + (Max% elongation/100)).

### 4.3 RNA extraction, library preparation, sequencing, read mapping to the reference genome and gene coverage reporting

RNA was isolated from HUVECs after exposure to cyclic stretch of 6% or not (0%). Total RNA was extracted using the NucleoSpin RNA II kit (Macherey-Nagel) according to the manufacturers’ instructions. cDNA libraries were constructed by the genomic platform of the University of Geneva (https://www.ige3.unige.ch/) using the Illumina TruSeq RNA sample preparation kit according to the manufacturers’ protocol. Libraries were sequenced using single- end (50 nt-long) on Illumina HiSeq2000 (Fig. 1B). FastQ reads were mapped to the ENSEMBL reference genome (GRCh38.96) using STAR version 2.4.0j [17] with standard settings, except that any reads mapping to more than one location of the genome (ambiguous reads) were discarded (*m* = 1).

### 4.4 RNA-seq data analysis

A unique gene model was used to quantify reads per gene. Briefly, the model considers all annotated exons of all annotated protein coding isoforms of a gene to create a unique gene where the genomic region of all exons are considered coming from the same RNA molecule and merged together. All reads overlapping the exons of each unique gene model were reported using featureCounts version 1.4.6-p1 [18]. Gene expression levels were reported as raw counts and in parallel normalized in Read per kilobase of exon per million reads mapped (RPKM) in order to filter out genes with low expression value (1 RPKM) before calling for differentially expressed genes. Library size normalizations and differential gene expression calculations have been performed using the package edgeR [19] designed for the R software [20]. Only genes having a significant fold change≥2 and the Benjamini-Hochberg corrected *p*-value<0.05 were considered for the differentially expressed genes analysis (Fig. 1B).

### 4.5 Gene set enrichment analysis (GSEA)

All annotated pathways for Homo sapiens, Mus musculus, Rattus norvegicus, Danio rerio, Sus scrofa and Saccharomyces cerevisiae available on WikiPathways database (http://www.wikipathways.org/index.php/WikiPathways) were used to generate gene sets, as well as the Kyoto Encyclopedia of Genes and Genomes metabolic pathways (KEGG http://www.genome.jp/kegg/) relative to GRCh38.96. Genes were ranked by their calculated fold changes (decreasing ranking). A gene set analysis using the GSEA package Version 2.2 [21,22] from the Broad Institute (MIT, Cambridge, MA) was used to analyse the pattern of differential gene expression between the two groups. Gene set permutations were performed 1000 times for each analysis. The normalized enrichment score (NES) was calculated for each gene set. GSEA results with a nominal false discovery rate (FDR) <0.05 and absolute normalized enrichment score (abs(NES))>1 were considered significant.

### 4.6 Reverse transcription and quantitative PCR

Total RNA concentration was determined using a NanoDrop 2000 (ThermoFisher Scientific). cDNA was synthesized from equal amounts of RNA using QuantiTect Reverse Transcription Kit (Qiagen) and qPCR was performed using ABI StepOne Plus detection system. We used Taqman gene expression assays (Applied Biosystems). The primers for *GJA1* (Hs00748445_s1), *GJA4* (Hs0074917_s1), *GJA5* (Hs00270952_s1) and *18S* (Hs03003631_g1) from ThermoFisher Scientific were used. All reactions were normalized to 18S.

### 4.7 Protein quantification and Western blot

Cell cultures were rinsed with PBS (pH=7.4) and lysed in RIPA buffer (50 mmol/L Tris–HCl pH=8, 30 mmol/L NaCl, 1% NP40, 10 mmol/L NaF, 2 mmol/L Na_3_VO_4,_ 1 mmol/L phenylmethylsulfonyl fluoride, complete protease inhibitor cocktail (Roche Applied Science), 1 mmol/L EDTA pH=7.4, 0.05% sodium dodecyl sulfate, and 5 mmol/L sodium-deoxycholate). Cell lysates were gently mixed at 4 °C for 20 min, and then spun at 13'500 rpm for 20 min to collect the supernatant. The concentration of the isolated proteins was determined using BCA Protein Assay Reagent (ThermoScientific). Ten µg of protein were separated by SDS-PAGE and electrophoretically transferred to PVDF membranes (Immobilon, Millipore). After 2 h blocking with 5% milk and 1% Tween in PBS, the membranes were incubated with primary antibodies recognizing phosphorylated eNOS at Serine 1177 (9571s, Cell Signaling, 1/1000), eNOS (610297, BD Transduction 1/2500), Cx43 (3512s, Cell Signaling, 1/1000), glyceraldehyde 3-phosphate dehydrogenase (GAPDH, MAB374, Millipore, 1/30000) or beta-actin (A5316, Sigma, 1/10000). Thereafter, the appropriate secondary horseradish peroxidase-conjugated antibodies (Jackson ImmunoResearch; 1/5000) were used, followed by ECL detection (Millipore) using ImageQuant LAS 4000 software. Band intensities were quantified using the NIH Image software (NIH AutoExtractor 1.51; National Institutes of Health).

### 4.8 Statistical analysis

qPCR and Western blot analyses were done with GraphPad Prism 9.4.1 software. Results are shown in mean±SEM. Comparisons of means have been performed using a ratio paired *t*-test. Data were considered statistically significant at *p* < 0.05.

## Limitations

We specifically examined the influence cyclic circumferential stretch on endothelial cell function. While our investigation contributes greatly to our understanding of the role of CCS, it may not fully replicate the EC response to the complex physiological environment present *in vivo* within intracranial aneurysms. Another limitation of our study is the use of HUVECs, which may not exhibit identical responses to cyclic circumferential stretch compared to endothelial cells in cerebral arteries.

## Ethics Statement

We confirm that the authors have read and followed the ethical requirements for publication in Data in Brief and that the current work does not involve human subjects, animal experiments, or any data collected from social media platforms.

## CRediT authorship contribution statement

**Mannekomba R. Diagbouga:** Validation, Formal analysis, Investigation, Visualization, Writing – original draft. **Sylvain Lemeille:** Visualization, Formal analysis, Data curation, Writing – original draft. **Sandrine Morel:** Conceptualization, Visualization, Writing – original draft. **Brenda R. Kwak:** Conceptualization, Writing – original draft, Funding acquisition, Supervision.

## Data Availability

GSE222834 (Original data) (GEO) doi:10.17632/wkxnrfbzzc.2 (Original data) (Mendeley Data)
